# Constant Transmission Properties of Variant Creutzfeldt-Jakob Disease in 5 Countries

**DOI:** 10.3201/eid1810.120792

**Published:** 2012-10

**Authors:** Abigail B. Diack, Diane Ritchie, Matthew Bishop, Victoria Pinion, Jean-Philippe Brandel, Stephane Haik, Fabrizio Tagliavini, Cornelia Van Duijn, Ermias D. Belay, Pierluigi Gambetti, Lawrence B. Schonberger, Pedro Piccardo, Robert G. Will, Jean C. Manson

**Affiliations:** The Roslin Institute, Easter Bush, Scotland, UK (A.B. Diack, V. Pinion, J.C. Manson);; University of Edinburgh, Edinburgh, Scotland, UK (D. Ritchie, M. Bishop, R.G. Will);; Cellule Nationale de Référence des Maladies de Creutzfeldt-Jakob, Universite Pierre et Marie Curie-Paris 6, INSERM, and CNRS, Paris, France (J.-P. Brandel, S. Haik);; Fdazione IRCCS Istituto Neurologico Carlo Besta, Milan, Italy (F. Tagliavini);; Erasmus University Medical School, Rotterdam, the Netherlands (C. Van Duijn);; Centers for Disease Control and Prevention, Atlanta, Georgia, USA (E.D. Belay, L.B. Schonberger);; Case Western Reserve University, Cleveland, Ohio, USA (P. Gambetti);; and Food and Drug Administration, Rockville, Maryland, USA (P. Piccardo)

**Keywords:** Prion, strain, variant Creutzfeldt-Jakob disease, transmissible spongiform encephalopathy, Creutzfeldt-Jakob disease, transmission properties, CJD, vCJD, TSE, BSE, prions, United States, the Netherlands, France, Italy, United Kingdom, USA, UK

## Abstract

Variant Creutzfeldt-Jakob disease (vCJD) has been reported in 12 countries. We hypothesized that a common strain of agent is responsible for all vCJD cases, regardless of geographic origin. To test this hypothesis, we inoculated strain-typing panels of wild-type mice with brain material from human vCJD case-patients from France, the Netherlands, Italy, and the United States. Mice were assessed for clinical disease, neuropathologic changes, and glycoform profile; results were compared with those for 2 reference vCJD cases from the United Kingdom. Transmission to mice occurred from each sample tested, and data were similar between non-UK and UK cases, with the exception of the ranking of mean clinical incubation times of mouse lines. These findings support the hypothesis that a single strain of infectious agent is responsible for all vCJD infections. However, differences in incubation times require further subpassage in mice to establish any true differences in strain properties between cases.

## Abstract 2

Current diagnostic criteria should be sufficient to detect new cases of vCJD.

Variant Creutzfeldt-Jakob disease (vCJD) is an acquired transmissible spongiform encephalopathy (TSE), or prion disease, that results in a fatal neurodegenerative condition in humans. vCJD was first reported in the United Kingdom in 1996 (*1*) and was most likely caused by dietary exposure to contaminated products from cattle that had bovine spongiform encephalopathy (BSE). The similarity between BSE and vCJD was shown by experimental transmission of the 2 diseases into standard panels of inbred wild-type mouse lines (RIII, C57BL, and VM [*1**,**2*]) and into FVB mice (*3*).

The strain properties of vCJD and BSE have been extensively characterized in these sets of mice by using a combination of the order in which each strain of mouse dies of disease (incubation period rankings), distribution of brain vacuolation at the terminal stage of disease (lesion profiles), distribution pattern of abnormal prion protein (PrP^Sc^) in the brain, and glycosylation pattern of PrP^Sc^ as assessed by Western blot analysis. BSE and vCJD produce highly reproducible and similar incubation period rankings and neuropathology, which indicates that they are the same strain in the RIII, C57BL, and VM mice, whereas in the FVB mice, both diseases exhibit the same pattern of PrP^Sc^ deposition (*3*–*5*).

During 1996–2011, a total of 176 cases of definite or probable vCJD were reported in the United Kingdom; the number of deaths peaked at 28 in 2000. Since 2006, deaths from vCJD have leveled off to 2–5 per year (*6*). Although vCJD was originally restricted to the United Kingdom, 49 cases have been reported in 11 other countries, including some outside Europe, bringing the worldwide total number of cases to 225. Most cases outside the United Kingdom have occurred in France, which is believed to be related to the large volume of beef imports; ≈60% of UK beef exports to Europe were destined for France (*7*). This possible relationship is borne out by a peak in vCJD cases in France 5 years after a similar peak in cases in the United Kingdom, which fits in well with the timing of an increase in beef imports from the United Kingdom during 1985–1995 (*7*).

More recently, comparative studies of vCJD cases from France and the United Kingdom have shown evidence from clinical, epidemiologic, pathologic, and biochemical analyses that a common strain of agent may be responsible for vCJD infection in both countries (*8*). In Europe, active surveillance to monitor BSE cases was implemented in 2001 (*6*,*9*). Although it appears that exports of meat, cattle, or both from the United Kingdom may have played a major role in the incidence of vCJD cases in other countries (*10*), indigenous BSE from before 2001 or another unidentified source may have caused some vCJD infections.

To determine whether vCJD cases in different countries have been caused by the same infectious agent, samples from 4 vCJD case-patients from the Netherlands, Italy, France, and the United States were made available for strain typing analysis using a standard panel of wild-type mouse lines. Each sample was methionine homozygous at codon 129 of the prion gene (Table). None of the patients had received blood or organ donations. Two patients (from Italy and the United States) were treated with quinacrine. All case-patients showed classic clinical characteristics of vCJD; postmortem examination confirmed the diagnosis. We conducted transmission studies in mice using brain samples from these 4 vCJD case-patients and compared transmission characteristics of all 4 cases to those of 2 historical cases of vCJD in the United Kingdom.

**Table:** Demographic and clinical features of case-patients with variant CJD from the Netherlands, France, Italy, and United States and 2 reference case-patients from the United Kingdom*

Characteristic	The Netherlands	France	Italy	United States	United Kingdom	
					1	2
Case-patient sex	F	F	F	F	M	M
Case-patient age at illness onset, y	24	36	25	22	24	35
Case-patient age at death, y	26	37	27	24	25	36
Disease duration, mo	19	14	27	32	14	12
Early psychiatric symptoms	Yes	Yes	Yes	Yes	Yes	Yes
Persistent painful sensory symptoms	Yes	No	Yes	No	Yes	No
Ataxia	Yes	Yes	Yes	Yes	Yes	Yes
Myoclonus, dystonia, or chorea	Yes	Yes	Yes	Yes	Yes	Yes
Dementia	Yes	Yes	Yes	Yes	Yes	Yes
No typical appearance of sporadic CJD on EEG	Yes	Yes	No	Yes	Yes	Yes
Bilateral symmetric pulvinar high signal on MRI scan of brain	Yes	No	Yes	Yes	Yes	No
Positive tonsil biopsy result	ND	Yes	Yes	Yes	ND	ND
Treatment	No specific treatment	No specific treatment	Quinacrine	Quinacrine	No specific treatment	No specific treatment
History of travel to or residence in the United Kingdom	No	No	No	Yes†	Yes	Yes
Codon 129MM	Yes	Yes	Yes	Yes	Yes	Yes
Type 2B PrP	Yes	Yes	Yes	Yes	Yes	Yes

*CJD, Creutzfeldt-Jakob disease; EEG, electroencephalogram; MRI, magnetic resonance imaging; ND, not done; PrP, prion protein. †Born in United Kingdom in 1979, moved to United States in 1992.

## Materials and Methods

### CJD Inocula

Frozen brain tissue consisting of ≈3 g of frontal cortex from each of 4 vCJD case-patients originating from the Netherlands, Italy, France, and the United States was available for transmission studies (Table). An additional similar sample of cerebellum from the vCJD case-patient from Italy was also studied to assess any differences in transmission characteristics between different regions of the brain. Tissue samples were homogenized at 10% (wt/vol) concentration in sterile physiologic saline and stored at −20°C until use. Ethical consent for the use of these materials for research was obtained and approved by the Lothian National Health Service Board Research Ethics Committee (reference: 2000/4/157).

### Experimental Animals

Two lines of mice expressing *Prn-p^a^* (RIII, C57BL) and 1 line expressing *Prn-p^b^* (VM) were used for the transmission experiments. The *Prn-p^a^* and *Prn-p^b^* alleles have a major influence on the incubation period of disease, with each TSE strain having a distinct and reproducible incubation period ranking with each of the *Prn-p* genotypes (*11*). Mice were anesthetized with halothane and inoculated with brain homogenate by a combination of intracerebral (i.c.) (0.02 mL) and intraperitoneal (0.1 mL) routes to ensure efficient uptake of the agent. Because the quantity of material available was limited, mice received only i.c. inoculations (0.02 mL) from the case-patient from the United States.

Mice were scored weekly for signs of clinical neurologic disease from 100 days as described by Fraser and Dickinson (*12*). Mice were killed by cervical dislocation whether due to TSE or other nonspecific disease and the brain removed at postmortem. Brains were cut sagitally; half was snap-frozen for biochemical analysis, and the remaining half was fixed in 10% formol saline for histologic analysis. Experiments were approved by The Roslin Institute’s Ethical Review committee and were conducted according to the regulations of the UK Home Office Animals (Scientific Procedures) Act 1986.

### Scoring of Vacuolation

After fixation, mouse brains were treated for 1.5 hours in 98% formic acid to reduce the infective titer for safety reasons. Brains were then cut transversely into 4 sections (hind brain, midbrain, forebrain, and hippocampus/thalamus) and embedded in paraffin. Samples underwent hematoxylin and eosin staining, and each mouse was scored for degree of vacuolation (ranked 1–5, with 1 the least affected) found in 9 standard gray matter regions and 3 white matter regions. Scores for >6 mice were averaged to produce a mean lesion profile for each of these areas (*12*).

### PrP Immunohistochemical Analysis

Paraffin-embedded tissue sections were pretreated to aid antigen retrieval by autoclaving for 10 min at 121°C. Sections were then immersed in formic acid for 10 min to enhance PrP labeling. PrP immunostaining was performed by using the monoclonal anti-PrP antibody 6H4 (Prionics, Schlieren-Zurich, Switzerland) at a dilution of 1:10,000 overnight at room temperature. The secondary antibody was biotinylated rabbit anti-mouse (Jackson ImmunoResearch Laboratories, Inc., West Grove, PA, USA) used at 1:400 for 1 h. The Vectastain Elite ABC Kit (Vector Laboratories, Burlingame, CA, USA) was used, and visualization of antibody binding was through deposition of 3,3′-diaminobenzidine chromogen.

### Western Blot Analysis of PrP^Sc^

Frozen brain samples were homogenized in 0.9% saline solution to yield a 10% suspension. This material was cleared by centrifugation and the supernatant treated with 50 µg/mL proteinase K for 1 h at 37°C (for detailed methods, see Head et al. [*13*]). The digested product was denatured and then loaded onto a 10% Bis/Tris NuPAGE Novex gel (Invitrogen, Paisley, UK). After electrophoresis, the gel was blotted onto polyvinylidene fluoride membranes. Detection of prion protein used the enhanced chemiluminescence ECL+ technique (Amersham Biosciences, Little Chalfont, UK) with primary antibody 6H4 at 1:30,000 and an anti-mouse IgG peroxidase-linked secondary antibody (Amersham Biosciences) at 1:60,000. Images were captured on radiographic film.

## Results

**Figure 1:**
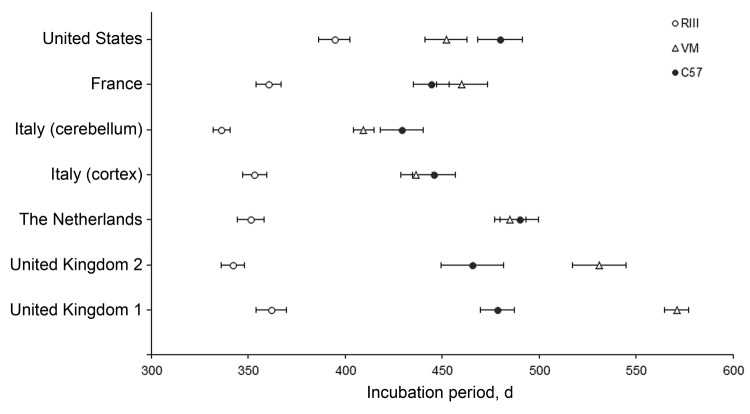
Comparison of variant Creutzfeldt-Jakob disease incubation periods from 5 sources in wild-type mice. Data show mean incubation period ± SEM. i.c., intracerebral; i.p., intraperitoneal; UK, United Kingdom.

All 5 vCJD brain isolates transmitted successfully to the wild-type mouse panel with the appearance of clinical and pathologic signs associated with prion disease. Mean incubation periods and mouse line ranking order were calculated and compared with reference data from the United Kingdom. The inocula from the Netherlands, Italy (cortex and cerebellum), and the United States showed variation in the temporal order of appearance of clinical symptoms in each of the mouse strains when compared with the UK reference cases (Figure 1). These 3 brain inocula showed the shortest incubation periods in the RIII mice, occurring at ≈365 days postinoculation (dpi), followed by VM at ≈450 dpi; the longest was found in the C57BL mice (≈465 dpi). The ranking of incubation time differed with brain inocula from France, with the C57BL mice dying of disease before the VM mouse line, similar to the pattern observed in the UK reference cases. The exact timing of the appearance of clinical symptoms varied, with wide ranges observed for each line of mouse within and between the different inocula; incubation periods were 301–463 days in RIII mice, 361–553 days in VM mice, and 335–553 days in C57BL mice.

### Lesion Profiles

**Figure 2:**
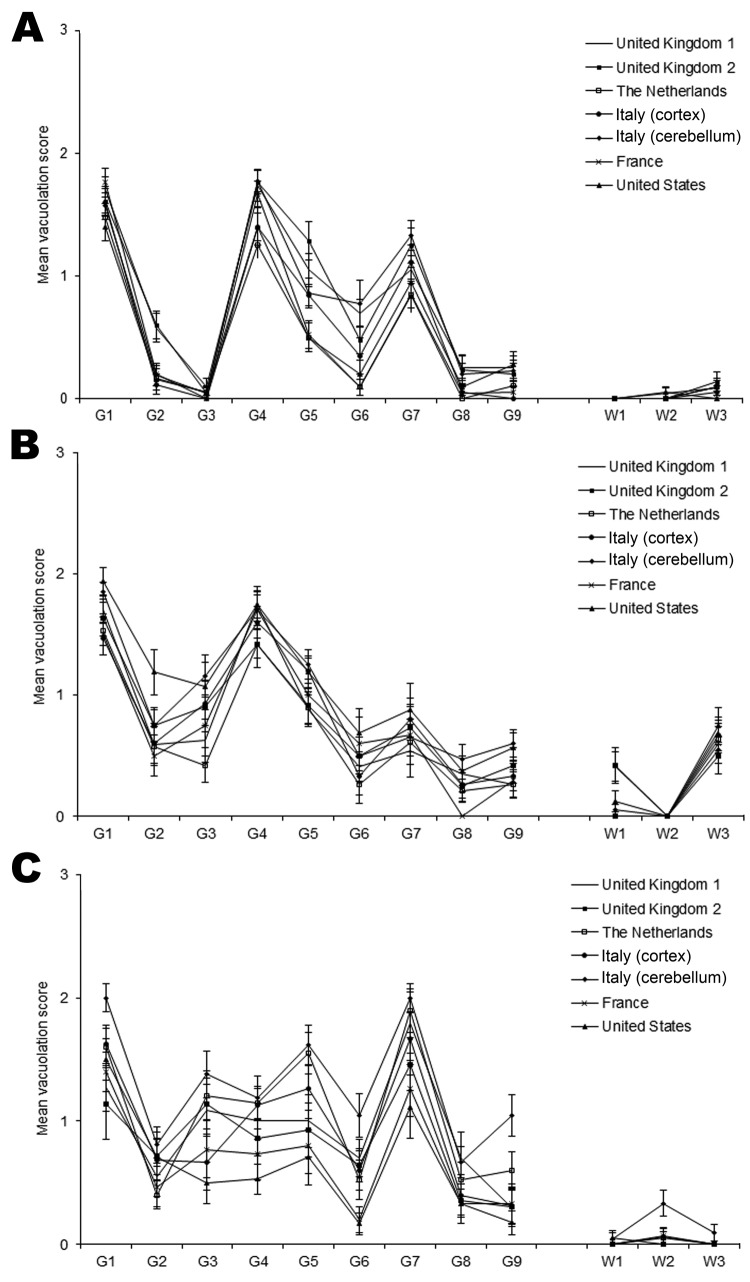
Lesion profile comparison of variant Creutzfeldt-Jakob disease cases show similarities in vacuolar pathology levels and regional distribution in mouse brains. Wild-type mouse lines RIII (A), C57 (B), and VM (C) are shown. Data show mean lesion profile ± SEM (n>6). G1–G9, gray matter scoring regions: G1, dorsal medulla; G2, cerebellar cortex; G3, superior colliculus; G4, hypothalamus; G5, thalamus; G6, hippocampus; G7, septum; G8, retrosplenial and adjacent motor cortex; G9, cingulate and adjacent motor cortex. W1–W3, white matter scoring regions: W1, cerebellar white matter; W2, mesencephalic tegmentum; W3, pyramidal tract.

Sufficient numbers of wild-type mice scored positive for the presence of TSE-associated vacuolation to enable lesion profiles to be generated from their mean scores. Similar patterns of vacuolation distribution were seen for all vCJD isolates, including those taken from either the cortex or cerebellum (case-patient from Italy) (Figure 2). Mouse strains of the same PrP genotype showed close similarities in lesion profiles with *Prn-p^a^* mice (RIII and C57BL), showing moderate gray matter vacuolation of the medulla, hypothalamus, and septum, with C57BL mice additionally exhibiting mild white matter vacuolation of the basal cerebellar peduncle. *Prn-p^b^* mice (VM) showed mild to moderate gray matter vacuolation of the medulla, superior colliculus, thalamus, and septum. Some variability was found in the intensity of vacuolation dependent on the vCJD isolate, most notably in the VM mice and particularly in the superior colliculus, hypothalamus, and thalamic regions.

### Immunohistochemical Analysis of PrP

**Figure 3:**
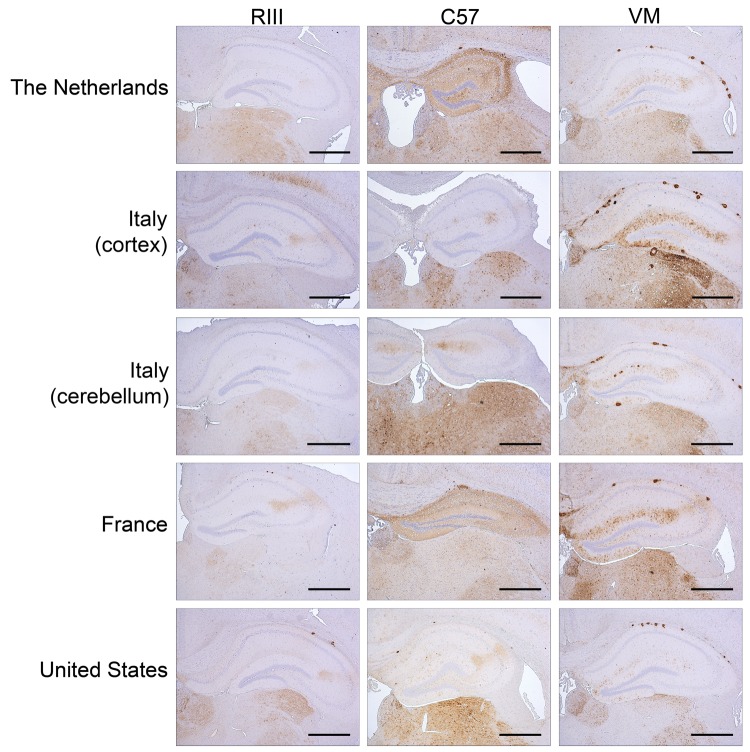
Immunohistochemical detection of abnormal prion protein (PrP^Sc^) in the hippocampus and thalamus of RIII, C57, and VM wild-type mice after inoculation with variant Creutzfeldt-Jakob disease brain tissue. Scale bars = 500 µm. The anti–prion protein detection antibody used was 6H4.

A total of 9 mice per inoculum, corresponding to 3 animals per genotype, were analyzed in a blinded experiment design. Animals from each group showed variability in the amount of PrP accumulation (Figure 3). Fine punctate deposits were the most consistently observed pattern of PrP accumulation in mice from all genotypes. However, in numerous instances, PrP plaques were also seen in the corpus callosum and subventricular area and, eventually, in the brain parenchyma of the cerebrum and cerebellum. VM mice showed a similar pattern of PrP deposition with each inoculum and consistently showed PrP plaques. RIII mice appeared to show less staining with the accumulation of PrP forming fine punctate, coarse, pericellular, and plaque deposits. In the C57BL line, PrP deposition appeared more variable within and between inocula.

### Biochemical Analysis of PrP

**Figure 4:**
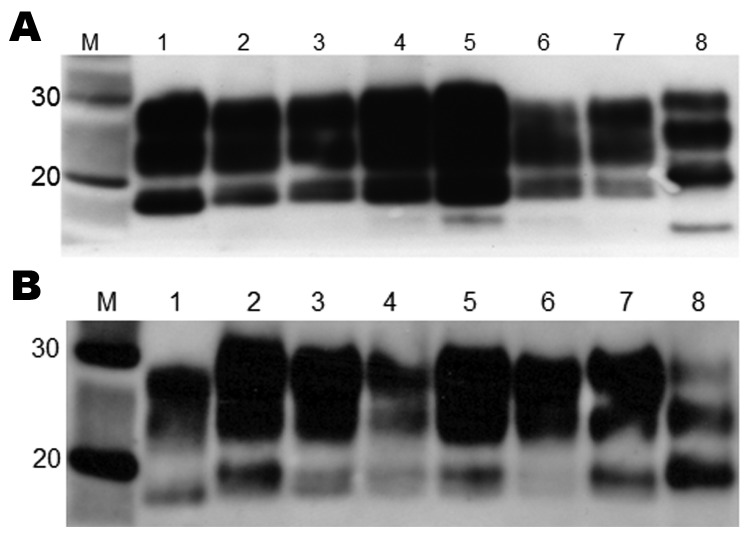
Western blot analysis of brain extracts from RIII (A) and VM (B) wild-type mice inoculated with variant Creutzfeldt-Jakob disease (vCJD) brain tissue. Lane M, positive control; lane 1, human vCJD brain homogenate (UK origin) showing the typical abnormal prion protein (PrP^Sc^) type 2B; lane 2, United Kingdom; lane 3, The Netherlands; lane 4: Italy (cortex); lane 5, Italy (cerebellum); lane 6, France; lane 7, United States; lane 8, human sporadic Creutzfeldt-Jakob disease brain homogenate showing the typical PrP^Sc^ type 1. Type 2B and 1 differ in mobility of the unglycosylated band (≈19 kDa and ≈20 kDa, respectively). All samples were treated with proteinase K. The anti–prion protein detection antibody used was 6H4.

Biochemical analysis of PrP^Sc^ by Western blot produced a type 2B–like gel mobility and glycosylation profile in all mouse strains (Figure 4). This profile is characterized by the predominance of the diglycosylated form of PrP^Sc^ and an ≈19-kDa unglycosylated fragment, similar to that seen in all vCJD human brain tissue examined to date. The unglycosylated fragment appeared as a doublet with a distinct upper and lower band and the relative intensity of the 2 bands varying with mouse *Prn-p* genotype; the lower band appeared more intense in *Prn-p^a^* mice and the higher band more intense in *Prn-p^b^* mice. The biochemical profile was identical for all 5 brain isolates and identical to the pattern observed in the UK cases.

## Discussion

We used transmission studies with wild-type mice to define the strain characteristics of geographically distinct cases of vCJD to establish whether a common strain of agent is responsible for vCJD cases from 5 different countries. Transmission properties such as TSE-associated vacuolation, PrP^Sc^ deposition, glycosylation profile, and mobility of PrP all show strong similarities between vCJD cases from the Netherlands, Italy, France, and the United States and with reference cases from the United Kingdom.

The distribution of TSE vacuolation and PrP^Sc^ deposition in the brains of the RIII and VM mice was similar for each brain isolate examined and indicates that the same strain of agent is present in each inoculate. C57BL mice showed more variability in PrP^Sc^ deposition, which may be a result of the transmission of the vCJD agent across a species barrier. Each brain isolate produced a type 2B–like glycoform profile on biochemical analysis; similar to results from previous studies, the unglycosylated fragment appeared as a doublet (*14*).

We observed differences in the timing of the onset of clinical signs of disease for each of the non-UK cases, which may be because of differences in the infectious titer of the different vCJD isolates. We also identified a change in the incubation period ranking of the mouse strains between the inoculum from France and other locations (Figure 1). For each brain isolate, the RIII mice were the first line to die of disease, consistent with previous transmissions from vCJD cases in the United Kingdom.

Furthermore, our study has shown that the ≈100-day difference in incubation period between the RIII and C57BL mice (both *Prn-p^a^*), which is characteristic of the BSE strain, has been maintained in the experimental transmission of the vCJD worldwide cases (*15*). The C57BL and VM mice have resulted in close incubation period ranges that can overlap, as was demonstrated previously (*14*,*15*). For inocula from the non-UK cases, incubation periods appeared in the order RIII, VM, C57BL, whereas the inocula from France showed incubation periods in the order RIII, C57BL, VM, as do the historic UK cases. This change in the order could be attributed to genetic drift in the mouse lines used over the course of the historical and current studies or variations in the strain of TSE agent.

The VM mice used in this study have shown substantially shortened incubation periods compared with those from in earlier studies, which may have caused the alteration in ranking. This alteration in incubation time ranking was also identified recently in a comparable transmission study involving a more recent UK vCJD case (M. Bishop et al., unpub. data). Moreover, the difference in time ranking is unlikely to be attributable to variation in brain area; we have established similar transmission characteristics between brain regions and have aimed in this study to use identical brain regions for each non-UK case. Differences in inoculation route are also not an explanation for the alteration in ranking. Differences were not found between the case from the United States, which was inoculated i.c. only, and the cases from the Netherlands and Italy, which were inoculated i.c. and intraperitoneally.

The infectivity titer of the inoculum may have played a role in alteration of the incubation periods. However, Ritchie et al. (*14*) suggested that changes in titer would affect all mouse strains and would not result in a change in the ranking of the mouse strains. Experimental transmission of vCJD from the case from the United States, in which lower volumes were inoculated, shows the same temporal pattern as the cases from the Netherlands and Italy.

Variability in incubation time is often associated with the primary transmission of a TSE agent between species but often stabilizes on subsequent mouse-to-mouse passage, while variability within groups decreases. Therefore, variations observed in this study do not necessarily point to strain variation between the cases of vCJD. However, to rule out this possibility, further transmission studies will be conducted. Moreover, passage of cases of vCJD occurring during the past 15 years will be undertaken in a comparative study to establish whether genetic drift in mouse lines or strain variation in vCJD underlies the differences observed in this study. Two of the brain isolates inoculated (from Italy and the United States) were from patients who had been treated with quinacrine. However, this treatment appears to have had no effect on incubation periods, vacuolation profiles, PrP^Sc^ deposition patterns, or the glycosylation and mobility of PrP.

The similarities in lesion profiles, biochemistry, and immunohistochemistry between this series of vCJD transmission studies support the hypothesis that a single strain of infectious agent is responsible for all vCJD cases, regardless of geographic origin, which would suggest that current diagnostic criteria for vCJD are sufficient to detect cases in all countries at this time. Still to be determined is whether the differences in incubation period rankings in some cases represent changes in strain phenotype over time, which could affect future diagnosis.
